# Identifying common disease trajectories of Alzheimer’s disease with electronic health records

**DOI:** 10.1002/alz.090812

**Published:** 2025-01-09

**Authors:** Mingzhou Fu, Timothy S Chang, UCLA Precision Health Data Discovery Repository Working Group

**Affiliations:** ^1^ David Geffen School of Medicine, University of California, Los Angeles, Los Angeles, CA USA; ^2^ Movement Disorders Programs, Department of Neurology, David Geffen School of Medicine, University of California, Los Angeles, Los Angeles, CA USA

## Abstract

**Background:**

Alzheimer’s disease (AD), a leading cause of dementia, poses a growing global public health challenge. While recent studies have identified AD risk factors, they often focus on specific comorbidities, neglecting the complex interrelations and temporal dynamics. Our study addresses this by analyzing AD progression through longitudinal trajectories, utilizing clinical diagnoses over time. Using machine learning and network analysis, we created a computational framework to identify common AD progression patterns.

**Method:**

We analyzed patient diagnoses from UCLA Health’s Electronic Health Records, coded with the International Classification of Diseases, version 10 (ICD‐10). Using the Fine and Gray model to detect significant temporal risk factors between diagnoses, we examined associations between diagnosis pairs and refined the patients' diagnostic trajectories, eliminating irrelevant disease transitions and delineating all possible trajectory combinations. These refined trajectories were compared using Dynamic Time Warping and grouped into clusters with hierarchical clustering. We investigated common AD trajectories through network analysis and compared patient demographics, AD manifestations, and genetic profiles across clusters. The Greedy Equivalence Search algorithm helped infer causal relationships within these trajectories.

**Result:**

Our analysis included 6,969 eligible AD patients, narrowing down to 1,486 patients with 3,372 unique AD progression trajectories after refinement. We identified four trajectory clusters: 1) a mental disorder cluster centered around major depressive disorder (MDD) (e.g., anxiety disorder → MDD) (N_patient = 525); 2) a cerebrovascular disease cluster (e.g. hypertensive crisis → other cerebrovascular diseases) (N_patient = 348); 3) a neurodegenerative disease cluster characterized (e.g., Parkinson’s disease → other degenerative disease of nervous system) (N_patient = 742); and 4) an outlier cluster predominantly involving external causes like infectious diseases (N_patient = 7). Significant differences were observed in race‐ethnicity, age at AD onset, disease duration, and the polygenic risk score (PRS) for AD across clusters. For example, patients in the cerebrovascular disease cluster exhibited higher mean AD PRS compared to other clusters (p = 0.05). Causal analysis indicated that 56.1% (47 out of 91) of the identified trajectory connections were causal.

**Conclusion:**

We uncovered AD diagnosis trajectories, incorporating temporal aspects and causal relationships. These insights could improve understanding of AD development, aid in subtype classification, and enhance risk assessment, which could significantly benefit patient care and medical research.

